# Genomic comparisons of *Brucella *spp. and closely related bacteria using base compositional and proteome based methods

**DOI:** 10.1186/1471-2148-10-249

**Published:** 2010-08-13

**Authors:** Jon Bohlin, Lars Snipen, Axel Cloeckaert, Karin Lagesen, David Ussery, Anja B Kristoffersen, Jacques Godfroid

**Affiliations:** 1 Norwegian School of Veterinary Science, Department of Food Safety and Infection Biology, Epicenter, Ullevålsveien 72, PO Box 8146 Dep, NO-0033 Oslo, Norway; 2 Norwegian University of Life Sciences, Department of Chemistry, Biotechnology and Food Sciences, Ås, Norway; 3 INRA, UR1282, Infectiologie Animale et Santé Publique, IASP, Nouzilly, F-37380, France; 4 University of Oslo, Department of Informatics, Pb. 1080, 0316 Oslo, Norway; 5 Center for Biological Sequence Analysis, Department of Systems Biology, Comparative genomics unit, Technical University of Denmark, DK-2800 Lyngby, Denmark; 6 National Veterinary Institute, Section for epidemiology, Pb 750 Sentrum, N-0106 Oslo, Norway; 7 Norwegian School of Veterinary Science, Department of Food Safety and Infection Biology, Section of Arctic Veterinary Medicine, Stakkevollveien 23, 9016 Tromsø, Norway

## Abstract

**Background:**

Classification of bacteria within the genus *Brucella *has been difficult due in part to considerable genomic homogeneity between the different species and biovars, in spite of clear differences in phenotypes. Therefore, many different methods have been used to assess *Brucella *taxonomy. In the current work, we examine 32 sequenced genomes from genus *Brucella *representing the six classical species, as well as more recently described species, using bioinformatical methods. Comparisons were made at the level of genomic DNA using oligonucleotide based methods (Markov chain based genomic signatures, genomic codon and amino acid frequencies based comparisons) and proteomes (all-against-all BLAST protein comparisons and pan-genomic analyses).

**Results:**

We found that the oligonucleotide based methods gave different results compared to that of the proteome based methods. Differences were also found between the oligonucleotide based methods used. Whilst the Markov chain based genomic signatures grouped the different species in genus *Brucella *according to host preference, the codon and amino acid frequencies based methods reflected small differences between the *Brucella *species. Only minor differences could be detected between all genera included in this study using the codon and amino acid frequencies based methods.

Proteome comparisons were found to be in strong accordance with current *Brucella *taxonomy indicating a remarkable association between gene gain or loss on one hand and mutations in marker genes on the other. The proteome based methods found greater similarity between *Brucella *species and *Ochrobactrum *species than between species within genus *Agrobacterium *compared to each other. In other words, proteome comparisons of species within genus *Agrobacterium *were found to be more diverse than proteome comparisons between species in genus *Brucella *and genus *Ochrobactrum*. Pan-genomic analyses indicated that uptake of DNA from outside genus *Brucella *appears to be limited.

**Conclusions:**

While both the proteome based methods and the Markov chain based genomic signatures were able to reflect environmental diversity between the different species and strains of genus *Brucella*, the genomic codon and amino acid frequencies based comparisons were not found adequate for such comparisons. The proteome comparison based phylogenies of the species in genus *Brucella *showed a surprising consistency with current *Brucella *taxonomy.

## Background

The genus *Brucella *belongs to the α-Proteobacteria order and consists of mostly intra-cellular bacteria that are known to be pathogenic in a wide range of mammal hosts [1]. The ailments caused by the different species and strains from genus *Brucella *are known collectively as brucellosis [1]. Brucellosis is a contagious zoonotic disease known to affect many different mammals ranging from livestock and humans to a wide variety of marine mammals. Each species or strain, however, has a narrow host range [1].

The *Brucella *genus has traditionally been classified into six species: *B. melitensis*, *B. suis*, *B. abortus*, *B. neotomae*, *B. ovis*, and *B. canis*, which are reflective of host preference. In 1985, it was proposed that the six *Brucella *species should be grouped as biovars of a single species based on DNA-DNA hybridization studies [2]. The *Brucella *Taxonomic Subcommittee of the International Committee on Systematics of Prokaryotes adopted this proposition. However, the international community of *Brucella *researchers has never accepted this change and a return to the pre-1986 taxonomy was advocated and eventually adopted by the *Brucella *Taxonomic Subcommittee [3]. Genus *Brucella *has been further expanded with a set of recently discovered species. Such species include *B. ceti *and *B. pinnipedialis *that have been isolated from cetaceans and pinnipeds [4]. *B. microti *has been isolated from the common vole [5], and *B. inopinata *was isolated from a breast implant infection in an woman with clinical signs of brucellosis [6].

The genomes sequenced from genus *Brucella *are also known to be very similar in terms of both base composition and genome size [1]. All sequenced species have a GC content of approximately 57%, and most genomes consist of approximately 3.3 Mbp divided on two chromosomes (see Table 1). None of the sequenced members of the *Brucella *genus have any plasmids reported [http://www.ncbi.nlm.nih.gov/genomes/lproks.cgi].

**Table 1:** 0^th ^order Markov chain model based cluster groups of *Brucella *genomes

Name	Accession	Database	Group	%GC	Size (mbp)	Host
*Brucella abortus *2308 (biovar 1)	AM040264.1	Genbank/NCBI	1	57	3.28	Cattle
*Brucella abortus *2308 A^§^	VBI00022-VBI00023	PATRIC	1	57	3.31	Cattle
*Brucella abortus *9-941 (biovar 1)	AE017223.1	Genbank/NCBI	1	57	3.28	Cattle
*Brucella abortus *S19 (biovar 1)	CP000887.1	Genbank/NCBI	1	57	3.29	Cattle
*Brucella canis *ATCC 23365	CP000872.1	Genbank/NCBI	1	57	3.32	Dog
*Brucella inopinata *BO1^§^	VBI00041-VBI00043	PATRIC	1	57	3.37	Human
*Brucella melitensis *16M (biovar 1)	AE008917.1	Genbank/NCBI	1	57	3.32	Sheep, goat
*Brucella melitensis *63/9 (biovar 2) ^§^	ACEM01000000	Broad Institute	1	57	3.29	Sheep, goat
*Brucella melitensis *ATCC 23457 (biovar 2)	CP001488.1	Genbank/NCBI	1	57	3.28	Sheep, goat
*Brucella ovis *ATCC 25840	CP000709.1	Genbank/NCBI	1	57	3.28	Sheep
*Brucella *sp. BO2^§^	VBI00103-VBI00105	PATRIC	1	57	3.28	Human
*Brucella suis *1330 (biovar 1)	AE014291.4	Genbank/NCBI	1	57	3.31	Pig
*Brucella suis *ATCC 23445 (biovar 2)	CP000911.1	Genbank/NCBI	1	57	3.31	Pig, hare
*Brucella ceti *B1/94^§^	ACEK01000000	Broad Institute	2	58	3.34	Dolphin, porpoise
*Brucella ceti *M13/05/1^§^	ACBP01000000	Broad Institute	2	58	3.34	Dolphin, porpoise
*Brucella ceti *M490/95/1^§^	ACEJ01000000	Broad Institute	2	58	3.35	Dolphin, porpoise
*Brucella ceti *M644/93/1^§^	ACBO01000000	Broad Institute	2	58	3.33	Dolphin, porpoise
*Brucella pinnipedialis *B2/94^§^	ACBN01000000	Broad Institute	2	58	3.34	Seal
*Brucella pinnipedialis *M292/94/1^§^	ACEF01000000	Broad Institute	2	58	3.37	Seal
*Brucella *sp. F5/99^§^	ACFF01000000	Broad Institute	2	58	3.4	Dolphin
*Brucella abortus *86/8/59 (biovar 2) ^§^	ACBJ01000000	Broad Institute	3	58	3.32	Cattle
*Brucella melitensis *Ether (biovar 3) ^§^	ACEI01000000	Broad Institute	3	57	3.28	Sheep, goat
*Brucella melitensis *Rev.1 (biovar 1)^§^	ACEG01000000	Broad Institute	3	57	3.31	Sheep, goat
*Brucella neotomae *5K33^§^	ACEH01000000	Broad Institute	3	58	3.33	Rodent
*Brucella *sp. 83/13^§^	ACBQ01000000	Broad Institute	3	58	3.29	Rodent
*Brucella suis *513 (biovar 5)^§^	ACBK01000000	Broad Institute	3	58	3.15	Pig
*Brucella suis *686 (biovar 3)^§^	ACBL01000000	Broad Institute	3	58	3.3	Pig
*Brucella pinnipedialis *M163/99/10^§^	ACBM01000000	Broad Institute	4	59	3.41	Seal
*Brucella abortus *292 (biovar 4)^§^	ACBH01000000	Broad Institute	5	58	3.28	Cattle
*Brucella abortus *870 (biovar 6)^§^	ACBG01000000	Broad Institute	5	58	3.27	Cattle
*Brucella abortus *C68 (biovar 9)^§^	ACEL01000000	Broad Institute	5	58	3.27	Cattle
*Brucella abortus *Tulya (biovar 3)^§^	ACBI01000000	Broad Institute	5	58	3.31	Human
*Agrobacterium radiobacter *K84	CP000628.1	Genbank/NCBI	6	60	6.66	Plant
*Agrobacterium tumefaciens *C58	AE007869.2	Genbank/NCBI	6	59	4.92	Plant
*Agrobacterium vitis *S4	CP000633.1	Genbank/NCBI	6	58	5.01	Plant
*Ochrobactrum anthropi *ATCC 49188	CP000758.1	Genbank/NCBI	6	56	4.78	Human, plant
*Ochrobactrum intermedium *LMG 3301^§^	VBI00028-VBI00031	PATRIC	6	58	4.73	Human

^§^Genomes not assembled; therefore GC content and genome size are only approximate values

The first *Brucella *species to be sequenced was *B. melitensis *16M (biovar 1) [7] followed closely by *B. suis *1330 (biovar 1) [8]. As more genomes are being sequenced, taxonomic classification of the *Brucella *genus is becoming more difficult, and many different methods have been applied [9]. The challenges involved in taxonomical classification of *Brucella *spp. are largely linked to the fact that marker genes typically used for phylogenetic classification are either missing or too similar to give any meaningful results [9,10]. Additionally, marker gene based methods like MLST and 16S rRNA do not directly reflect changes in gene content and may therefore fail to reproduce a broader view of the differences between species, strains, and biovars [10,11]. SNP analysis gives a better overview of changes happening at the genome level, but does not directly reflect changes in gene content [10]. Hence, taxonomic classification of *Brucella *spp. is a challenging task touching on difficult taxonomic and phylogenetic issues in prokaryotic species definition as a whole [9].

The aim of this study was to examine the strength and weaknesses of a set of methods for phylogenetic classification based on whole genome comparisons. This was carried out using a number of sequenced genomes from species and strains taken from genus *Brucella *and the closely related genus *Agrobacterium *and genus *Ochrobactrum. *This study was motivated by the genomic homogeneity and the difficult phylogenetic assessment of genus *Brucella*. Genomic comparisons were performed using a number of different methods that reflect changes at both the proteome level and the base composition level.

The comparison methods reflecting DNA composition used in this study include oligonucleotide based 0^th^, 1^st^, and 2^nd ^order Markov chain genomic signature models (ZOM, FOM, SOM, respectively) [12], and codon and amino acid frequencies analyses [13].

For the proteome based comparisons of the genomes, the Prodigal gene finder [14] was used to predict open reading frames (ORFs) in all genomes used in the study (See Table 1). Whole genome BLAST comparisons were subsequently performed between all proteomes, *i.e. *all-against-all gene comparisons between all genomes according to the guidelines given by Ussery *et al. *[15]. In addition, pan- and core genome analyses [16,17] were carried out to map gene exchange in sequenced members of genus *Brucella *and the closely related phylogenetic genera such as *Agrobacterium *and *Ochrobactrum *[18].

Scholz and co-workers [18] have carried out a thorough 16S rRNA analysis and we refer to that article for these results.

Of the methods described above, the Markov chain models and codon and amino acid frequencies based analyses best reflect base compositional differences and whole genome mutational bias [12,19]. The oligonucleotide based methods are sensitive to mutations at the genome level, and therefore share certain similarities with the whole genome SNP analyses conducted by Foster *et al. *[10]. The BLAST comparisons and pan-genomic analyses focus on gene content comparisons and gene exchange and may thus be considered as complementary to the oligonucleotide frequencies based methods that mirror base compositional differences. To the best of our knowledge, recent whole genome based gene comparisons of *Brucella *species, similar to the work conducted here, have only been carried out for 5 *Brucella *genomes (*B. ovis *ATCC25840, *B. suis *1330 (biovar 1), *B. abortus *9-941 (biovar 1), *B. melitensis *16M (biovar 1) and *B. abortus *2308 (biovar 1)) by Tsolis *et al. *[20]. In the present work however, we perform whole genome comparisons of 32 *Brucella *genomes (Table 1) using a variety of different genomic methods to obtain deeper insight into the obscure evolution of genus *Brucella*. In addition to the 32 *Brucella *genomes, we also include three sequenced genomes from genus *Agrobacterium*, *A. radiobacter *K84, *A. tumefaciens *C58, *A. vitis *S4, and two from genus *Ochrobactrum*, *O*. *anthropi *ATCC 49188 and *O*. *intermedium *LMG 3301, to examine the relative difference between these closely related microbes [18].

## Results

### Markov chain analyses

**Figure 1:**
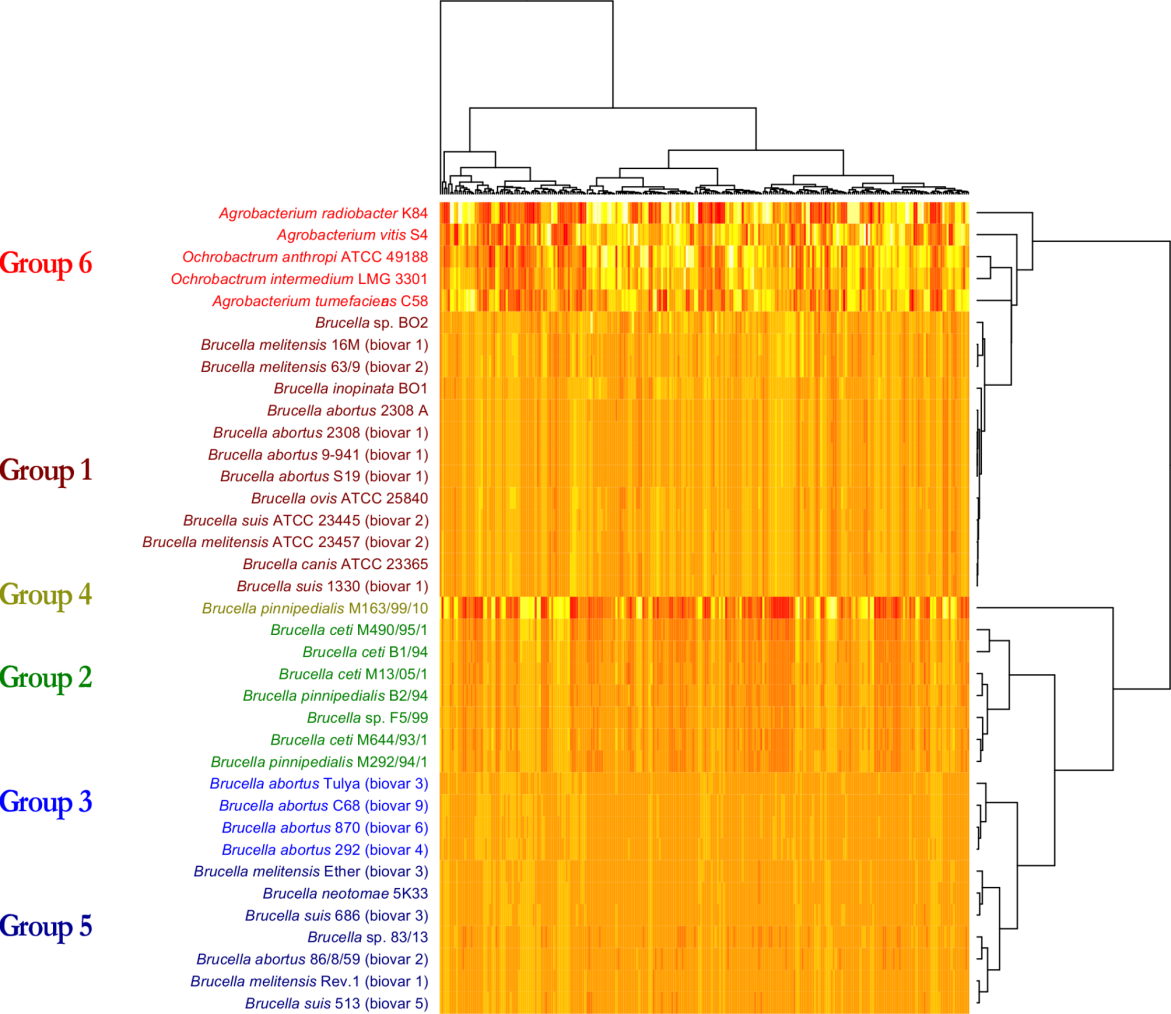
**ZOM based heatmap**. The ZOM Based heatmap shows all genomes compared using cluster analysis based on 0^th ^order Markov chain model predicted tetranucleotide frequencies. It can be seen that the sequenced species from genus *Brucella *are very similar in terms of tetranucleotide usage patterns, with larger differences found in the more distantly related genera of *Agrobacterium *and *Ochrobactrum*. Although all species in genus *Brucella *are very similar in terms of base composition, as measured using the ZOM based method, several subgroups can be observed. For instance, marine associated (Groups 2 and 4) and terrestrial mammal associated (Groups 1, 3 and 5) species of genus *Brucella *are segregated into different groups.

**Figure 2:**
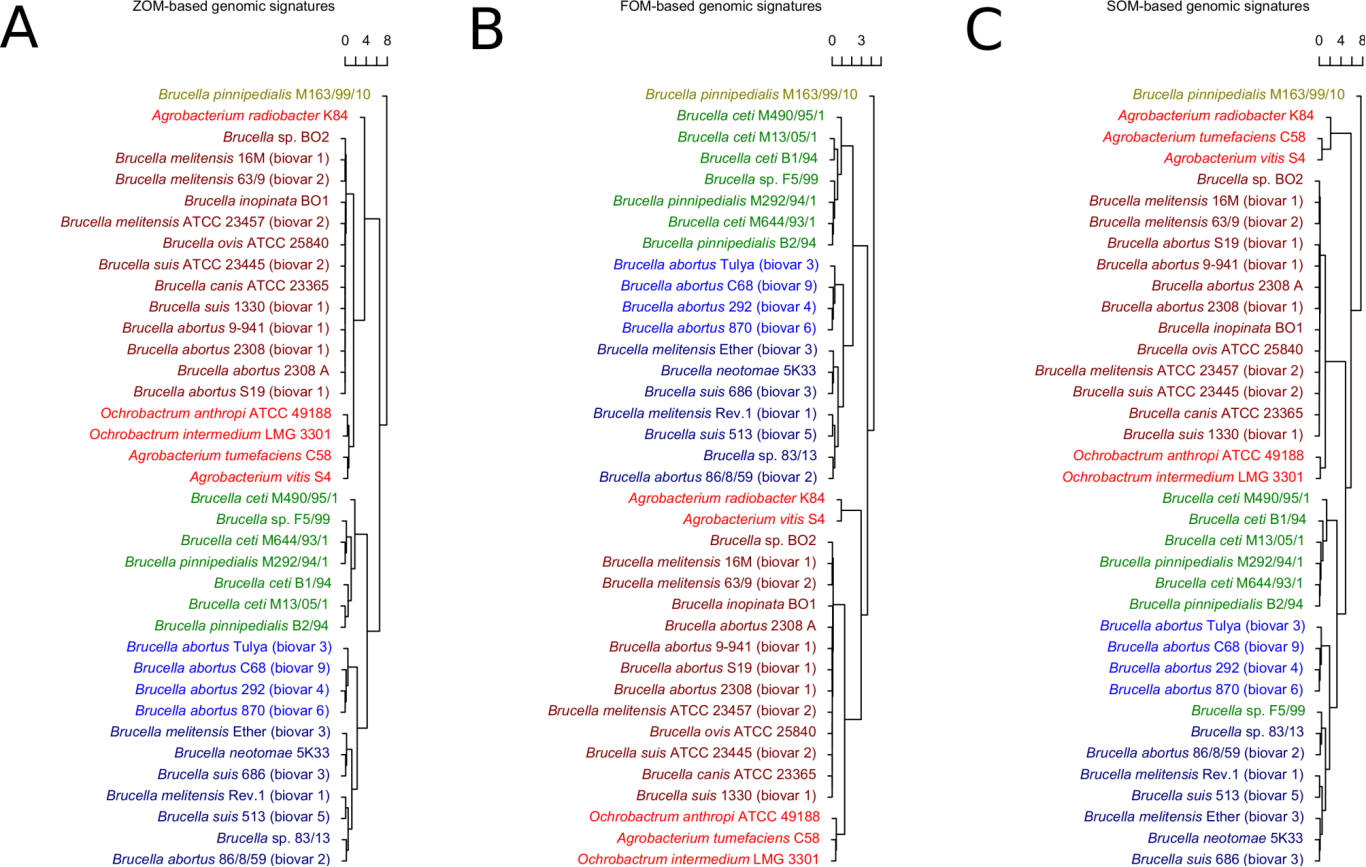
**Phylogenetic trees based on ZOM, FOM and SOM based genomic signatures**. The ZOM, FOM and SOM based trees (panels A, B, C, respectively) all show genomes compared using 0^th^, 1^st ^and 2^nd ^order Markov chain model based genomic signatures and clustered using average linkage with Euclidean distance. It can be seen that there is large agreement between the different Markov chain based genomic signatures. For all models, marine and terrestrial host based species of genus *Brucella *appear in distinctive groups, except for the SOM based tree (C) where the marine mammal associated strain *B. *sp. F5/99 is grouped with terrestrial mammal associated species. The more distantly related genera of *Agrobacterium *and *Ochrobactrum *appear in separate groups than the species of genus *Brucella *for all genomic signature based models.

The outcomes of the Markov chain model based genomic signatures analyses are shown in figures 1 and 2. Figure 1 shows a set of cluster groups obtained using the ZOM based heatmap. Table 1 describes these cluster groups in more detail. The ZOM, FOM and SOM based genomic signatures, which were used to produce the phylogenetic trees seen in Figure 2, are based on comparisons using the Pearson correlation method. The ZOM based heatmap seen in Figure 1 however, applies hierarchical clustering directly on vectors of the relative abundances of tetranucleotide frequencies (see the methods section for more details on these methods). In general, all Markov chain based models produced similar clusters, as can be seen from the color-coding in figures 1 and 2.

From Table 1 it can be seen that the ZOM based heatmap (Figure 1) was divided into 6 groups. Groups 1 and 3 consist of *Brucella *species associated with terrestrial mammals, while groups 2 and 4 contain exclusively marine mammal associated species. Figure 1 shows that the only species in group 4, *B. pinnipedialis *strain 163/99/10 isolated from a hooded seal (*Cystophora cristata*), shares many of the same tetranucleotide patterns with the species in group 2. Group 5 consists entirely of *B. abortus *strains; although similar in base composition to groups 1 and 3, group 5 appears to constitute a separate group. Group 6 is the most diverse group in terms of genome size, base composition, and GC content, and consists exclusively of non-*Brucella *species. Figure 2 shows that the species in the defined groups described in Table 1 were, for all Markov chain based genomic signatures, found in similar cluster groups with the exception of *B. *sp. F5/99 in group 2, which was isolated from a Pacific bottlenose dolphin (*Tursiops truncatus*) [21]. Although both ZOM- and FOM based methods cluster *B. *sp. F5/99 in the same group, the SOM method found the bacterium more closely related to cluster group 3.

All Markov chain based models placed *B. pinnipedialis *M163/99/10 in a separate group indicating relatively large genomic base compositional differences with the other *Brucella *species and strains (see Figure 1). *B. abortus *2308 (biovar 1) clustered more closely to the *B. abortus *9-941 (biovar 1) and *B. abortus *S19 (biovar 1) than the *B. melitensis *strains, implying that the Markov chain based models support the return to the pre-1986 taxonomy discussed above. In general, the Markov chain based genomic signatures found all members of cluster group 1 to be very similar in terms of base composition. From the viewpoint of genomic signatures, this implies that group 1 can in fact be considered as one phylogenetically coherent group. The same might be said for group 2. All Markov chain based models group the species from group 2 correspondingly; with *B. ceti *M490/95/1, isolated from harbour seal (*Phoca vitulina*), having a somewhat larger base compositional difference than the other genomes in the group. A notable exception is *B. *sp. F5/99 that was found more similar to group 3 (rather than group 2) for the SOM based comparison method. All Markov chain based genomic signatures group the strains in cluster group 5 similarly and the species from the genera *Agrobacterium *and *Ochrobactrum *cluster separately from genus *Brucella*. Group 6 is thus entirely made up of species from the genera *Ochrobactrum *and *Agrobacterium*, and contains no species from genus *Brucella*. In Figure 2, the species from *Agrobacterium *and *Ochrobactrum *are not found in one coherent group as in Figure 1. However, a closer inspection of Figure 2 reveals that all non-*Brucella *species clustered separately from the *Brucella *species. The cluster groups were found to be so different that no reliable conclusion could be made as to whether the species in genus *Agrobacterium *or genus *Ochrobactrum *were more similar to the species in genus *Brucella *as measured with the Markov chain based models.

The ZOM based heatmap (Figure 1) shows that there are relatively large base compositional differences between genus *Brucella *and the genera *Agrobacterium *and *Ochrobactrum*. However, the groups resulting from the ZOM based heatmap indicate that there are large similarities between the groups containing species from genus *Brucella*. The genomes found in cluster group 1 appear to be very similar in terms of base composition, with only negligible differences detected between some of the genomes. The heatmap in Figure 1 also indicates that *B. pinnipedialis *M163/99/10 may have diverged from cluster group 2. Additionally, the tetranucleotide patterns taken from the *B. pinnipedialis *M163/99/10 genome resemble the other species in cluster group 2, but are more pronounced. Cluster group 5 appears to be similar to cluster group 3 in terms of tetranucleotide relative abundance patterns although some subtle differences can be observed between the different species in the group.

### Codon and amino acid frequencies

**Figure 3:**
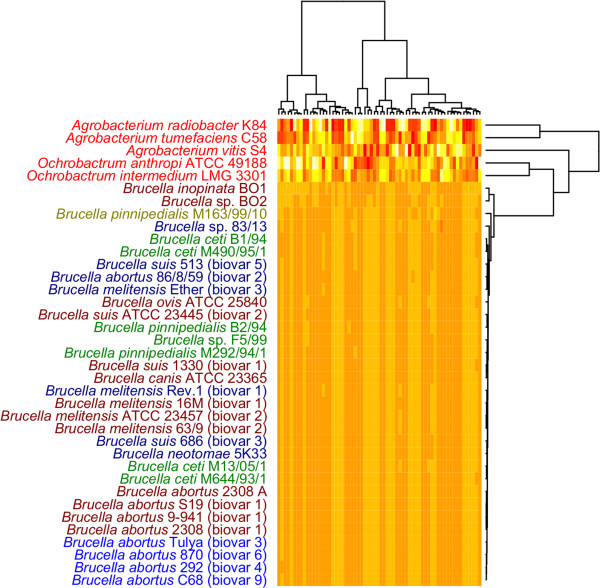
**Codon frequencies based heatmap**. The codon frequencies based heatmap is made from all the trinucleotide (codon) frequencies found in all predicted genes from the genomes of all species in the genera: *Brucella*, *Agrobacterium *and *Ochrobactrum*. It can be seen that there are small differences among the species in genus *Brucella *and only slightly more variance between the species found in the other genera.

**Figure 4:**
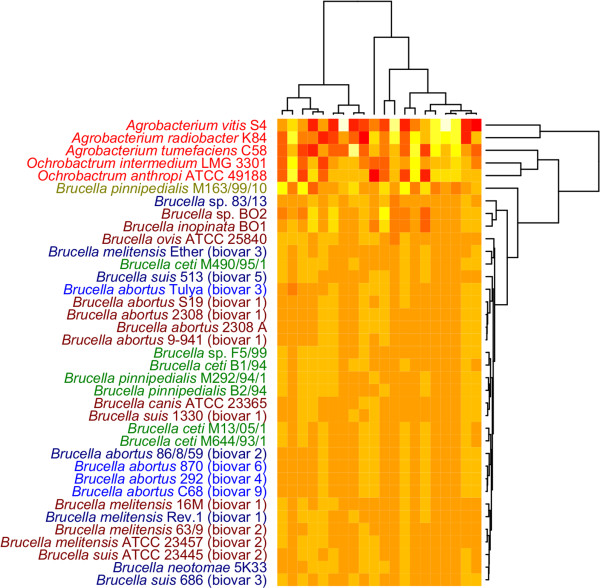
**Amino acid cluster diagram**. The amino acid frequencies based cluster diagram is made from the amino acid usage found in the predicted genes of the genomes in question. Amino acid frequencies correlate strongly with codon usage frequencies, but at a lower resolution. It can be seen that only small differences can be detected between species in genus *Brucella*.

The codon and amino acid frequencies based comparison methods (Figure 3 and 4) are similar to the Markov chain based models in that they are also based on oligonucleotide frequencies data. However, genomic codon and amino acid frequencies are more influenced by GC content than the Markov chain model based genomic signatures described above since no GC content or smaller oligonucleotide normalization is performed.

From Figure 3 it can be seen that the organisms making up genus *Brucella *form one homogeneous group with only minor frequency changes in a few of the codons. No distinction is made between terrestrial- and marine mammal associated *Brucella *species. The group of species not belonging to genus *Brucella *consists of more heterogeneous genomes in terms of codon frequencies. Figure 4 show the amino acid frequencies from the translated codon frequencies taken from the genomes of all organisms in the study. This heatmap appears to be more diverse than the codon frequencies based heatmap. However, the overall topology appears to be similar to that of Figure 3, with no distinction made between marine- and terrestrial mammal associated *Brucella *species. Hence, the general topology of the amino acid frequencies based heatmap appears to resemble the codon frequencies based heatmap. The resulting heatmaps from the amino acid and codon frequencies based comparisons stand in contrast to the ZOM based heatmap where a clear distinction between marine and terrestrial mammal host associated species from genus *Brucella *can be observed. The ZOM based heatmap appears to give a more detailed distinction between the different organisms as compared to the heatmaps based on both amino acid and codon frequencies. This is especially apparent in closely related species and strains, which are hardly distinguishable from the codon and amino acid frequencies based heatmaps. However, the codon and amino acid frequencies based cluster diagrams reinforce the impression obtained from the Markov chain models that the genomes of the species in genus *Brucella *have a somewhat different base composition from the species in genus *Agrobacterium *and genus *Ochrobactrum*.

### Proteome comparisons and the BLAST matrix

**Figure 5:**
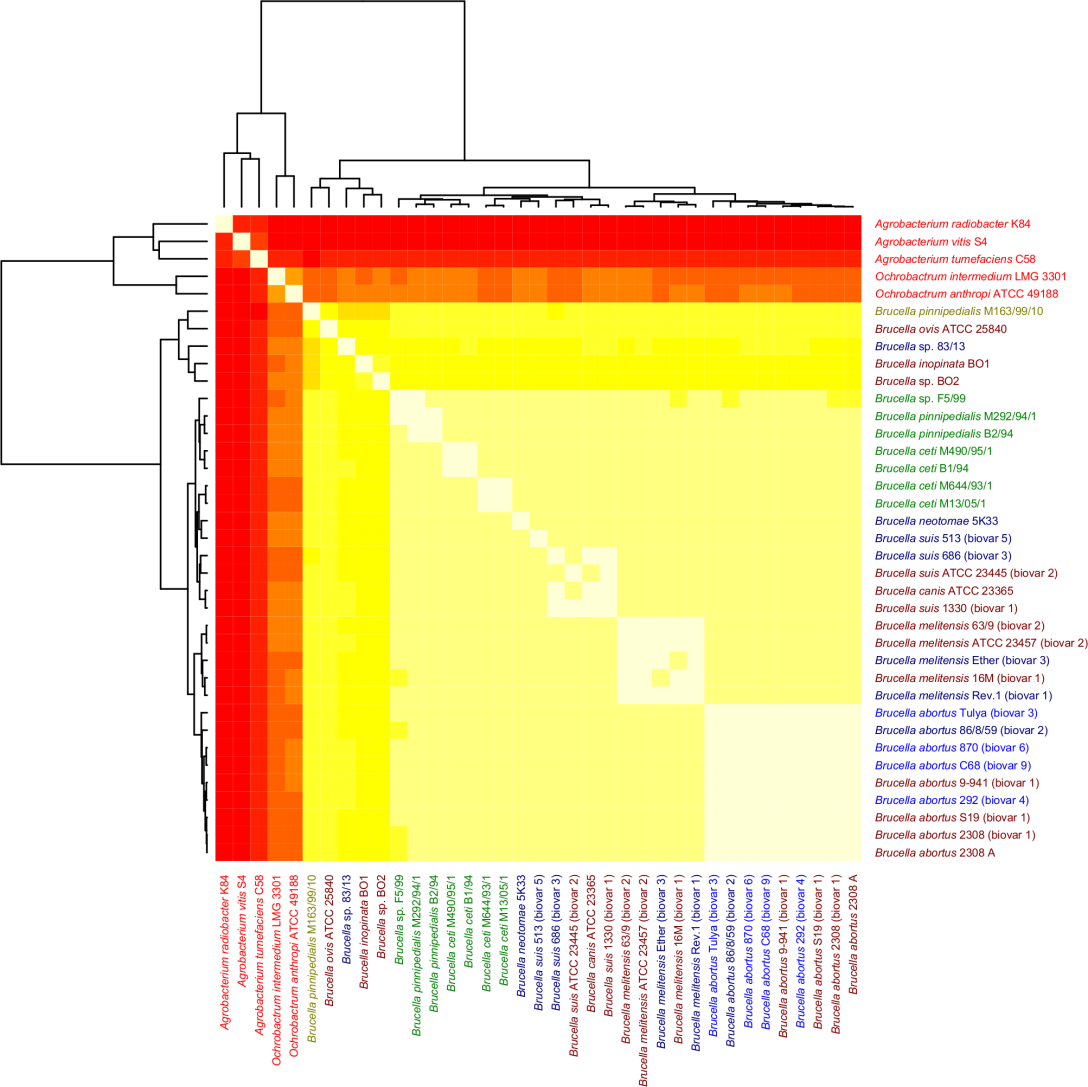
**BLAST matrix**. Genomes are compared gene-wise using BLAST. All genes were converted to proteins and compared pair-wise all-against-all for each genome. Lighter color means closer similarity. Paralogs are removed when genomes are compared to them self which means that the hit score is less than 100%.

The BLAST matrix in Figure 5 is based on all-against-all comparisons between the proteomes of all genomes discussed in the present work. More consistency, in terms of current *Brucella *taxonomy, was found in the BLAST matrix as compared to both the clusters based on the Markov chain based genomic signatures and codon and amino acid frequencies. This indicates that phylogenetic classification of genus *Brucella *based on marker genes, for instance multi locus sequence typing [22], show a surprising similarity to the organism's total gene content. Thus, at least for *Brucella *spp., there appears to be an association between mutations in marker genes and gene content.

The most similar species in genus *Brucella *in terms of gene content (or proteomes) were found to be *B. abortus *9-941 (biovar 1) and the vaccine strain *B. abortus *S19 (biovar 1) (99.8%, see additional file 1). *O. anthropi *ATCC 49188 was found to have a 48.3% proteome similarity with *B. suis *1330 (biovar 1), and was the closest match between a *Brucella *species and a non-*Brucella *species. The two species from genus *Ochrobactrum *shared 57% of their proteins, while the most similar proteomes between two species from genus *Agrobacterium*, *A. vitis *S4 and *A. tumefaciens *C58, shared only 35% of their genes. For the species in genus *Brucella*, the poorest match based on proteome comparisons was between *B. pinnipedialis *M163/99/10 and *B. inopinata *BO1 (70%). The most dissimilar proteomes all together, were *A. radiobacter *K8 and *B. pinnipedialis *M163/99/10 sharing only 21% of their genes. The most similar proteomes from the genera *Agrobacterium *and *Brucella *were *A. tumefaciens *C58 and *B. suis *1330 (biovar 1), respectively, sharing 29% of their proteomes.

### Pan-genome

Pan-genomic analysis is concerned with mapping genes that are conserved (shell) and variable (cloud) among closely related organisms, usually within a genus or species [17].

**Figure 6:**
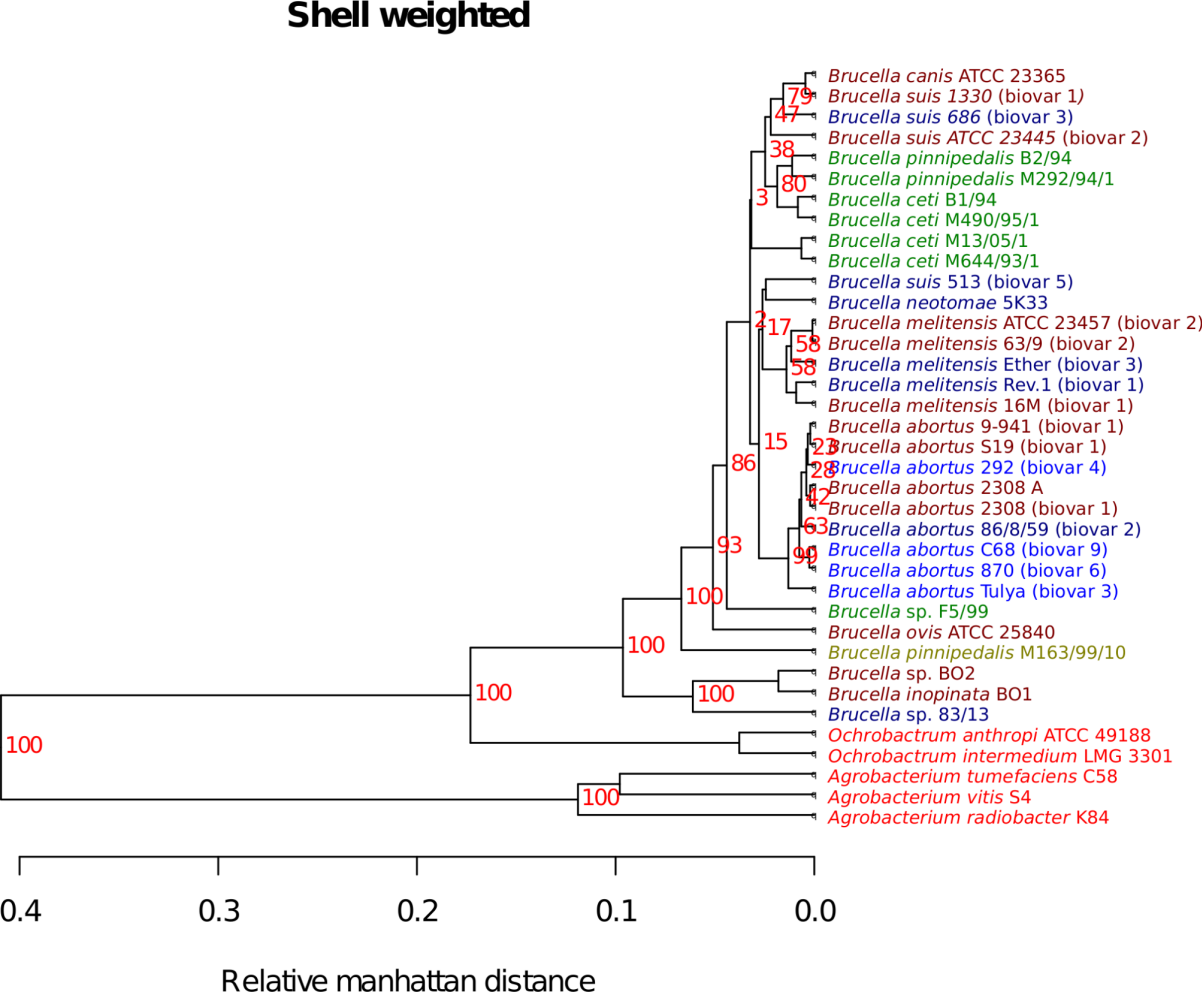
**Pan-genomic shell tree**. The Figure shows a pan-genomic tree based on a weighting strategy emphasizing 'shell' genes. The red numbers are bootstrap-values, in percentage, based on 100 bootstrap samples.

**Figure 7:**
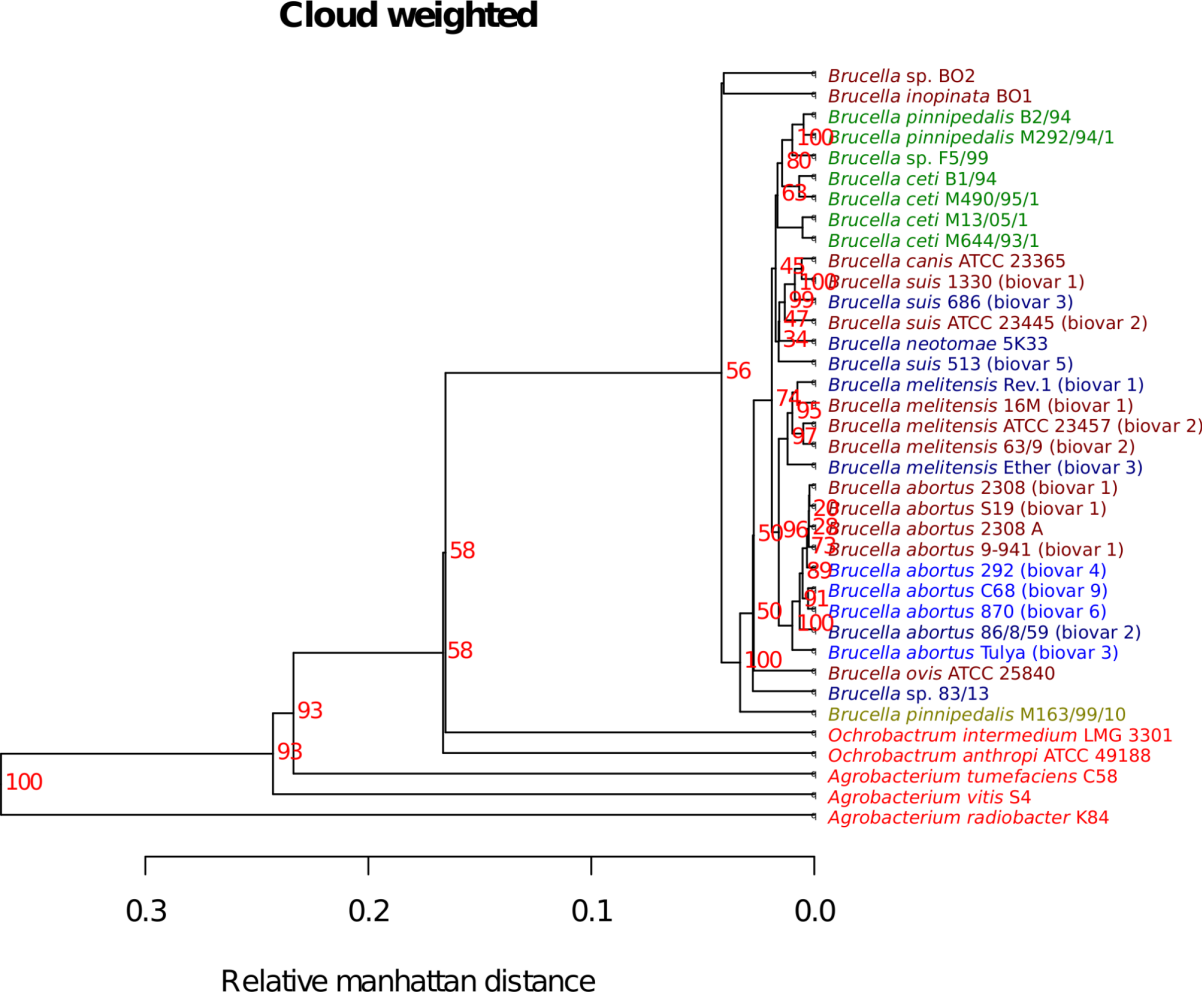
**Pan-genomic cloud tree**. The Figure shows a pan-genomic tree based on a weighting strategy emphasizing 'cloud' genes. The red numbers are bootstrap-values, in percentage, based on 100 bootstrap samples.

Two *Brucella *pan-genome trees are shown in figures 6 and 7. One emphasizing the shared shell genes (Figure 6) and the other the less conserved cloud genes (Figure 7). The shell genes are frequently observed in the pan-genome, and differences in shell-gene content most likely reflect an evolution over a longer time span [17]. The slow divergences of orthologs have for some strains led to the complete loss of gene families. This is manifested as the bigger differences in the shell-weighted pan-genome tree. The shell tree also shows that three strains: *B. *sp. BO2, *B. inopinata *BO1 and *B. *sp. 83/13 differ significantly from the others. Additionally, both trees show a remarkable difference in gene content between *B. pinnipedalis *M163/99/10 and the other strains of the same species. The *B. suis *513 (biovar 5) appears to be separated from the other *B. suis *strains, which may be indicative of a substantial difference in gene content. However, bootstrap support is low for most branches, which implies that the detected difference in gene content is negligible.

The cloud weighted tree (Figure 7), which is based on promiscuous genes, shows the same overall trend that is observed for the shell tree discussed above, even though the distances between genomes have changed. The cloud genes occur rarely and may be enriched with mobile elements such as inverted repeats, insertion sequences, and transposons making them difficult to isolate as distinctive genes. The fact that the cloud tree and the shell tree show the same topology can be seen as an indication that gene uptake from more distantly related organisms is rare in genus *Brucella*.

**Figure 8:**
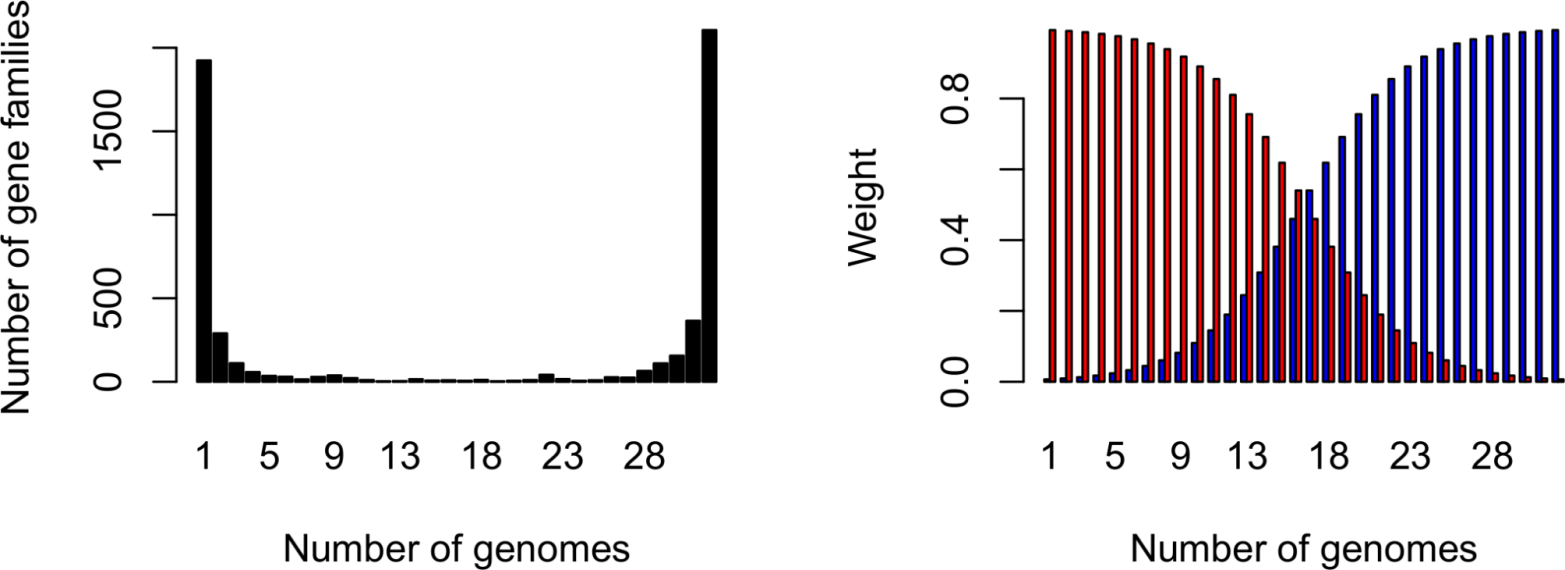
**Weighted pan-genomes**. The left panel is a bar-chart showing the number of gene families observed in 1, 2, ..., 32 gene families within the *Brucella *pan-genome (only genomes from species in genus *Brucella *are included). The gene families only found in a few genomes (left-wing bars) are called cloud genes, and those found in most genomes (right-wing bars) are called shell genes. The core genes are those that are found in all genomes (rightmost bar). The right panel indicates two different weighting strategies for computing the pan-genome tree. The red bars give more weight to cloud genes when comparing two genomes, while the blue bars emphasize the shell genes.

The pan-genomic analyses conducted here (figures 6, 7 and 8) reveal that there appears to be little genetic exchange within the sequenced species from genus *Brucella*. Compared to other bacteria, the present analyses of *Brucella *spp. uncovers greater homogeneity in terms of shared gene content than other species, such as *Streptococcus *spp. [16], *E. coli *spp.[23] and *Burkholderia *spp. [15]. In line with the BLAST matrix, the shell and cloud trees (figures 6 and 7, respectively) showed a remarkable consistency to current *Brucella *taxonomy. Only minor rearrangements were detected between the shell and cloud trees, with the departing of *B. *sp. F5/99 from the group of marine mammal associated species as the most notable exception. This may indicate that the sequenced genomes from genus *Brucella *are strongly conserved since differences in the potentially mobile genes described by the cloud tree are in accordance with the shell tree consisting of more conserved genes shared by all members in genus *Brucella*. Although DNA uptake from the environment and distantly related organisms occur in genus *Brucella *[24,25], it may be relatively rare otherwise it is expected that larger rearrangements would have been observed in the cloud tree [23].

## Discussion

### Markov chain based genomic signatures

The oligonucleotide frequencies based models discussed in the present work are not affected by genomic re-arrangements since estimations are based on oligonucleotide frequencies from all DNA sequences available. Differences between organisms, as measured using the oligonucleotide frequencies based methods, are therefore due to changes in base composition at the genome level caused first and foremost by mutational bias and to a lesser degree by possible horizontal transfer or DNA uptake [12]. Since the Markov chain based genomic signatures are normalized with respect to GC content and smaller oligonucleotides, they reflect a stronger degree of 'inertia' than the amino acid and codon frequencies based methods.

Our results, obtained using the Markov chain based genomic signatures, differ from the taxonomic assessments based on singular marker genes from multi locus sequencing [22]. This could be due to the low resolution of the Markov chain models or, alternatively, due to the genomic properties reflected by these methods. The ZOM based clusters have been shown to be sensitive to environmental conditions as well as phylogeny [26]. The reliability of methods based on equal probability of mutations throughout an entire genome, like all oligonucleotide based methods discussed here, must also be questioned. As sequencing of the genomes of organisms is increasing, more information is gained casting doubts about the validity of the molecular clock hypothesis [27].

### Comparisons based on codon and amino acid frequencies

The ZOM, codon frequencies and amino acid frequencies based cluster diagrams (figures 1,3 and 4) group the species in genus *Brucella *different than the gene based methods (figures 5, 6 and 7). This may indicate that conflicting evolutionary forces are driving mutational bias and gene content in genus *Brucella*. Codon and amino acid frequencies are remarkably similar for *Brucella *spp. compared to the species in the genera *Agrobacterium *and *Ochrobactrum*. The lack of consistency between the codon and amino acid frequencies based heatmaps on one side and the ZOM based heatmap on the other, may be due to a stronger phylogenetic signal found in the 0^th ^order Markov chain based model [28,29]. While the species in genus *Brucella *isolated from marine mammals formed one homogeneous group in the dendrograms produced by the Markov chain based genomic signatures, no such clustering could be detected with the codon and amino acid frequencies based heatmaps. Codon frequencies, which are highly affected by genomic GC content, will in turn affect amino acid frequencies [13].

### Evolutionary indications

Studies show that bacteria, which conform to an intracellular lifestyle, tend to become more AT rich [30,31]. Mutations have a tendency to go from G and C to A and T [32]. Mutations in genes will lead to genes that are non-expressible and eventually lost resulting in a reduced genome size [30]. AT content is associated with genome size in bacteria, where AT rich genomes tend to be smaller than GC rich genomes [33-35]. Table 1 shows that the marine mammal associated *Brucella *spp. are more GC rich than their terrestrial mammal based counterparts. In addition, assuming that the sizes of the non-assembled genomes are approximately correct, the marine mammal associated species in genus *Brucella *appear to have, on average, slightly larger genomes than the terrestrial mammal associated species. A possible scenario therefore, assuming a hypothetical ancestor X for both marine and terrestrial mammal *Brucella *spp., is that the marine mammal species of genus *Brucella *are closer to X than the terrestrial mammal associated species. One possible explanation for this is that the terrestrial mammal associated species have been living longer with their hosts than their marine mammal based relatives. The most GC rich genome of all *Brucella *spp. is the genome of *B. pinnipedialis *M163/99/10. It is therefore tempting to speculate that the hypothetical ancestor X could have a genome that is more similar to the marine mammal associated strain. The most AT rich genome from genus *Brucella *examined in this study is *B. ovis *ATCC 25840. From the perspective that intracellular bacteria tend to become more AT rich [30], it is possible that the genome of *B. ovis *ATCC 25840 is the least similar, in terms of base composition, to the hypothetical ancestor X of all species in genus *Brucella *examined here. It should be noted that the idea that *B. pinnipedialis *M163/99/10 is the last descendant from X rests on the assumption that genomes from intracellular bacteria become progressively smaller and more AT rich [25]. *Buchnera aphidicola*, *Mycobacterium leprae *and *Sodalis glossinidius *are examples of microbes all presumed to have adapted to an intra-cellular environment with the consequences of increased AT richness and genome reduction [30,36,37]. The cyanobacterium *Prochlorococcus marinus *MED4 provides an interesting example of a free-living bacterium, not associated with any host, becoming more AT rich and having undergone genome reduction possibly due to adaption to an environment with less nutrients available [38].

We emphasize again that the scenario described above is only one possible explanation. An alternative assertion to the genome reduction hypothesis is that species found in similar environments tend to have genomes with similar AT content regardless of phylogenetic relationship [39,40]. Therefore, the difference in AT content between the marine and terrestrial mammal *Brucella *strains may be a consequence of environmental differences.

### Gene based comparisons

The remarkable consistency between the proteome based comparisons and current *Brucella *taxonomy differs from the DNA based methods. Therefore, the congruent shell and cloud trees suggest that DNA uptake from species outside genus *Brucella *is an infrequent event, at least for the sequenced genomes included in this study. This is also supported by SNP analysis, which indicate that DNA exchange is fairly uncommon in genus *Brucella *[10]. Since the phylogeny recreated by the pan-genomic analyses resemble the pair-wise BLAST comparisons, it appears that gene similarity in the species of genus *Brucella *is concordant to current taxonomy based on marker genes and similar methods [9]. This implies that any mutation in the marker genes may be indirectly linked to gene gain or loss in genus *Brucella*. The difference in results obtained using the oligonucleotide based methods and the gene based methods suggest that the genomic properties reflected by the respective methods represent different perspectives. In previous work, we found that the ZOM based method is associated with a set of genomic properties including environment and phylogeny [26]. The ZOM based method is therefore a more composed method than the marker gene based comparison methods, in the sense that the ZOM heatmap topology is determined from a multitude of genomic properties.

The close resemblance found between the marker gene based phylogeny on one hand and the proteome based phylogeny on the other may thus be an indication that the mutations in the marker genes are somehow connected to gene loss or gain. In contrast to the result obtained with the Markov chain based genomic signatures, the differences in gene content in genus *Brucella *seem to be in accordance with a genome-wide molecular clock [41].

All species in genus *Agrobacterium *and genus *Ochrobactrum *are, in general, different from the species in genus *Brucella *both in terms of the oligonucleotide based methods and the gene based methods. The closest match in terms of gene content between a genome of a species from genus *Brucella *and a non-*Brucella *species was found to be between *B. suis 1330 *(biovar 1) and *O. anthropi *ATCC 49188, having a gene similarity of 48%. All genomes of the species in genus *Brucella *showed a gene content similarity of more than 70%. This raises again the question whether the genomes of the species in genus *Brucella *discussed in the present work should be divided into different species rather than strains. Such a scenario would imply that the members of genus *Ochrobactrum *would join genus *Brucella*, while the present species in genus *Brucella *would all be one species, but different subspecies or strains. For instance, the two genomes of the two species in genus *Ochrobactrum *discussed here were found to share only 57% of their genes, and the most similar genomes of genus *Agrobacterium *shared only 35% of their genes. Thus, based on the methods described in this work, there are many aspects that must be taken into consideration when taxonomy and phylogeny is to be decided.

## Conclusions

We find that the ZOM based heatmap is superior to both the codon and amino acid frequencies based heatmaps at distinguishing between closely related species and strains. The Markov chain based genomic signatures appear to have a higher resolution than the codon frequencies. The codon frequencies have a higher resolution than the amino acid frequencies. The amino acid frequencies between the genomes however, appear to be more diverse than the codon frequencies. Figure 2 shows that the differences between the different Markov chain models is small.

The proteome based comparisons, *i.e. *the BLAST matrix and pan-genomic analyses, differ somewhat from the base composition based methods, *i.e. *Markov chain based genomic signatures and codon and amino acid frequencies based methods, in terms of species classification. The BLAST matrix, based on pair-wise gene comparisons between the genomes of all microbes support the present *Brucella *taxonomy remarkably well suggesting a correlation between marker gene based phylogeny and the proteomes in genus *Brucella*. The pan-genomic analyses, including both shell and cloud weighted trees, also support the present *Brucella *taxonomy suggesting, in addition, infrequent horizontal transfer between species from genus *Brucella *and organisms belonging to other genera.

Comparing the pan-genomic trees to the base compositional based Markov chain models and codon and amino acid frequencies based methods, it can be concluded that subtle differences can be found below protein level. While there appears to be a fairly conserved gene pool in genus *Brucella*, more differences can also be found at the nucleotide level using the Markov chain based genomic signatures, which appeared to group the different species more strongly according to environment.

## Methods

All genomes were downloaded from Genbank [http://www.ncbi.nlm.nih.gov/genomes/lproks.cgi], the PATRIC website [42] [http://patric.vbi.vt.edu/] and the Broad Institute [http://www.broadinstitute.org/annotation/genome/brucella_group/MultiHome.html]. All genomes consisting of two chromosomes were concatenated into one file for each genome. All oligonucleotide based analyses were carried out in the 5'-3' direction for each genome (see [43,44] for justification of this). All statistical and mathematical analyses were carried out using the program R [45].

### Genomic signatures based on the 0^th^, 1^st ^and 2^nd ^order Markov chain models

The 0^th^, 1^st ^and 2^nd ^order Markov chain model based genomic signatures, referred to here respectively as ZOM, FOM and SOM based models, are different methods used to compare genomes by estimating the total difference between observed and approximated tetranucleotide frequencies. A thorough explanation of the different Markov chain model based genomic signatures can be found in [12]. Therefore, only a brief explanation of the notation used and a superficial introduction will be given below.

### The heatmap based on the 0^th ^order Markov chain model

This heatmap (Figure 1), referred to as the ZOM-heatmap, is based on pair-wise comparisons using the 0^th ^order Markov chain model ([12,26,44]). Tetranucleotide frequencies are estimated for all genomes and normalized with respect to the corresponding nucleotide frequencies:

(1) $$\rho_{XYZW}(f)=\frac{f_{XYZW}}{f_{X}f_{Y}f_{Z}f_{W}}$$

A vector, consisting of the relative abundances found using Equation (1) from all possible tetranucleotide combinations (4^4 ^= 256), is created for each genome. These vectors were clustered using average linkage hierarchical clustering with the Euclidean distance measure.

### Dendrograms based on 0^th^, 1^st ^and 2^nd ^order Markov property based genomic signatures

Tetranucleotide frequencies are approximated from genomic data according to the Markov chain based genomic signature model used. ZOM based genomic signatures approximate genomic tetranucleotide frequencies using genomic mononucleotide frequencies as described by Equation (1) above. FOM based signatures approximate genomic tetranucleotide frequencies using a combination of both genomic mono- and dinucleotide frequencies.

(2) $$\xi_{XYZW}(f)=\frac{f_{Y}f_{Z}f_{XYZW}}{f_{XY}f_{YZ}f_{ZW}}$$

SOM based genomic signatures approximate genomic tetranucleotide frequencies using genomic di- and trinucleotide frequencies:

(3) $$\eta_{XYZW}(f)=\frac{f_{XYZW}f_{YZ}}{f_{XYZ}f_{YZW}}$$

Instead of the average absolute distance measure used by Karlin and others [46-48] for pair-wise comparisons, we use the Pearson correlation measure for all signature based methods discussed above to compare two DNA sequences:

(4) $$\begin{array}{c} Cor_{\xi }(f,g)=\frac{\sum_{XYZW}[\left(\xi_{XYZW}(f)-\bar{\xi_{XYZW}(f)}\right)}{\sqrt{\sum_{XYZW}{\left(\xi_{XYZW}(f)-\bar{\xi_{XYZW}(f)}\right)}^{2}}}\times \\ \times \frac{\left(\xi_{XYZW}(g)-\bar{\xi_{XYZW}(g)}\right)]}{\sqrt{\sum_{XYZW}{\left(\xi_{XYZW}(g)-\bar{\xi_{XYZW}(g)}\right)}^{2}}} \end{array}$$

This formula gives the standard Pearson correlation coefficient between two DNA sequences *f *and *g*, using the FOM based genomic signature, *i.e. ξ*_*XYZW*_. Two identical sequences will result in a value of 1, while two completely different sequences will result in a value of 0. For instance, by comparing the DNA sequence of a bacterial genome to a completely random DNA sequence, with similar GC content, will result in a value very close to 0.

To create the phylogenetic trees, all genomes were compared pair-wise using the Pearson correlation measure (Equation (4)) with the ZOM, FOM and SOM based methods. The resulting ZOM, FOM and SOM correlation matrices, obtained from the pair-wise comparisons, are then clustered using hierarchical clustering. Average linkage was used as the clustering method to make the clustering as unbiased as possible. Because the difference between genomes, as measured using the Markov chain model based genomic signatures, was specified using the Pearson correlation coefficient, the Manhattan method was used as the distance measure.

### The codon and amino acid frequencies based heatmaps

The codon frequencies are based on overlapping trinucleotide frequencies in open reading frames predicted for all genomes. The open reading frames were predicted using the Prodigal gene finder [14]. Vectors of codon and amino acid frequencies, similar to the 0^th ^order Markov chain model discussed above, were calculated for every genome. The amino acid based heatmap was created using vectors containing amino acid frequencies from all converted open reading frames in each genome. The clustering method used for the frequency vectors of both codons and amino acids was identical to the clustering method used to generate the ZOM based heatmap, *i.e. *hierarchical clustering based on Manhattan distance and average linkage.

### The BLAST matrix proteome comparisons

Genes were predicted from the selected genomes using the Prodigal gene finder [14]. A BLAST matrix was constructed by performing pair-wise gene comparisons using BLAST for all genomes [15,49]. Based on these results, sequences were clustered into gene families according to the 'fifty-fifty' rule, *i.e*. two sequences are in the same family if the best local alignment between them cover at least 50% of the length of both sequences and also contain at least 50% identities ([16]). When a genome is compared to itself using BLAST paralogs are excluded. Thus, a genome compared to it self will seldom match 100% since the paralogs are not included.

### Pan-genomics

Gene families were computed as described above for the BLAST matrix. The presence or absence of gene families was stored as a pan-matrix *M*, where each row *i *corresponds to a gene family and each column *j *a genome. Then, if gene family *i *is present in genome *j*, *M*_*ij *_*= 1*, if not *M*_*ij *_*= 0*.

The presence or absence of pan-genome gene families can be used to give a high-resolution clustering of genomes. We have constructed a pan-genome tree based on relative Manhattan distances between genomes, computed from the pan-matrix. The distance between the genomes *l *and *k *is simply the fraction of gene families where their presence or absence status differs [50]. When computing this distance, some genes may be given less weight than others, *i.e. *disagreement in presence or absence status is more important for some types of genes [51]. When considering pan-genomes we propose the weightings shown in Figure 8. The shell genes are the gene families often observed among the genomes, while the cloud genes are rarely observed [52]. Both shell and cloud type of genes can be emphasized by the weighting strategies shown in Figure 8.

## Authors' contributions

JB wrote the paper and conducted the base compositional analyses; LS performed the pan-genomic analyses and together with KL carried out the protein based analyses. JB, AC and JG analyzed the data and related the findings to *Brucella *taxonomy. JB, LS and ABK carried out mathematical/statistical analyses. JB, AC, JG and DU analyzed the data, and drafted and revised the manuscript. The project was initiated by JG. All authors have read and approved the final manuscript.

## Supplementary Material

Additional file 1 **An Excel file containing the BLAST matrix showing percentage of proteome similarity resulting from the all-against-all comparisons of the genomes discussed in the study**. This percentage table was used to produce the heatmap in Figure 5. Click here for file
